# Barriers and Facilitators of Nurses' Involvement in Guideline Development and Adaptation: A Qualitative Evidence Synthesis Protocol

**DOI:** 10.1002/gin2.70044

**Published:** 2025-09-20

**Authors:** Linda Velapi, Sara Cooper, Tamara Kredo, Bey Schmidt

**Affiliations:** ^1^ School of Public Health University of the Western Cape Cape Town South Africa; ^2^ Cochrane South Africa, South African Medical Research Council Cape Town South Africa; ^3^ School of Public Health and Family Medicine University of Cape Town Cape Town South Africa; ^4^ Department of Global Health Division of Epidemiology and Biostatistics, Faculty of Medicine and Health Sciences Stellenbosch University Stellenbosch South Africa; ^5^ Health Systems Research Unit South African Medical Research Council Cape Town South Africa

## Abstract

**Introduction:**

Nurses often use clinical practice guidelines to assist them in treating childhood cases. Guidelines provide an easily accessible resource that clinicians can review to expand their knowledge base and determine patient care. Those involved in the guideline development and adaptation process usually have a better understanding when it comes to making use of the guideline. It is imperative to understand how the implementers of guidelines can be involved in the guideline development and adaptation process. The use of a Qualitative Evidence Synthesis is encouraged as it is the most appropriate method for this review because it enables the systematic integration of findings from primary qualitative studies that explore social, contextual, and experiential dimensions. This review seeks to answer the question: What are the views, experiences, and perceived barriers and facilitators of nurses involved in the development or adaptation of clinical practice guidelines? Using the SPIDER framework, this synthesis will focus on qualitative studies involving nurses (S), their participation in guideline development and adaptation (PI), using interviews or focus groups (D), to explore perceptions of engagement, challenges, and enabling factors (E), within a qualitative research paradigm (R).

**Objectives:**

To identify and synthesize studies exploring methods and processes for involving nurses in guideline development and adaptation. To explore the barriers to and facilitators of involving nurses in guideline development and adaptation.

**Methods:**

This review will follow the thematic synthesis approach and will be reported according to the ENTREQ checklist to ensure transparency in synthesis methods and the PRISMA guidelines for systematic reporting of the review process. Searches will be carried out in PubMed, CINAHL, Scopus, Medline and PsycINFO. We will apply thematic content analysis, which involves coding of extracted text, developing descriptive themes and generating analytical themes.

**Trial registration:** PROSPERO registration number: CRD42024580363.

## Summary

- This is the first qualitative review to focus specifically on nurses' involvement in developing and adapting clinical practice guidelines, addressing a significant gap in the literature.
- Using thematic synthesis, we will integrate findings from global qualitative studies to explore nurses' experiences, as well as the barriers and facilitators to their contributions in guideline processes.
- Our review will follow a transparent and rigorous methodology, guided by established frameworks (ENTREQ, CASP, CIS) to ensure the credibility and trustworthiness of our findings.
- The results will provide evidence‐based insights to strengthen nurse engagement in guideline development, improve interdisciplinary collaboration, and enhance the uptake of clinical guidelines.
- The findings have direct relevance for policy and practice, helping to ensure that guidelines are context‐sensitive, implementable, and aligned with the realities of frontline nursing.

## 1 Introduction and Background

### 1.1 Description of Topic

The death rate of children under the age of five has improved significantly over the last three decades [1]. While UNICEF applauds this global success, it is important to note that there is still a heavy burden of child deaths due to preventable or treatable causes such as pneumonia, malaria, and diarrhoea [1]. Target 3.2 of Sustainable Development Goal (SDG 3) of the 2030 agenda specifies the end of preventable deaths of newborns and children under 5 by lowering the neonatal and under‐5 mortality rates to at least 12 and 25 deaths per 1000 live births, respectively, by 2030 [2].

The majority of childhood diseases are initially seen presenting at primary health care sites, where nurses are the primary clinicians treating the cases. Nurses often use clinical practice guidelines to assist them in treating the children. According to Garbi [3], guidelines provide an easily accessible resource that clinicians can review to expand their knowledge base and determine patient care. This allows clinicians to stay up to date with current developments despite the rapid expansion of new information. Guerra‐Farfan et al. [4] also highlight that the benefit of using guidelines is that they promote evidence‐based practice. The disadvantage, however, is that guidelines may be developed by different professional groups (clinical specialty groups, government agencies, private organizations, policy makers, etc), each with their own goals and intended uses [4].

#### 1.1.1 Clinical Practice Guidelines

Clinical practice guidelines (from now on referred to as guidelines) are defined as statements that include recommendations intended to optimize patient care [5]. These are informed by a systematic review of evidence and are assessed for appropriateness of specific healthcare decisions, services, and outcomes. These documents are integral in guiding practice decision‐making, they are considered potentially beneficial for patients, healthcare professionals, and healthcare systems because they can improve health outcomes [5, 6].

#### 1.1.2 Guideline Development

In the past 20 years, the rate of guideline development has risen exponentially. Guidelines are perceived to present the best evidence for managing clinical situations, which include conditions or symptoms, and are upheld as the gold standard of high‐quality healthcare [7]. However, inconsistencies have been identified in the principles underpinning guideline development and the processes leading to best practice recommendations. Currently, best practice methods entail both technical processes (searching and appraising evidence‐based research) and social processes (which refer to the contextual activities such as expert consensus‐building, stakeholder engagement and collaboration, and local adaptation, that are necessary to translate evidence into practical, relevant and context‐sensitive recommendations). The steps include recognizing the priority needs and identifying the scope of the guideline, recruitment of an interdisciplinary working group and engaging with key stakeholders, searching for evidence, developing best practice recommendations, external review and consultation, dissemination and implementation of recommendations [3, 8]. To contribute towards the successful implementation of the guidelines, adapting the recommendations to the local situation is pivotal for its success.

#### 1.1.3 Guideline Adaptation

Customizing a guideline to a particular context may improve acceptance and adherence (context could range from a single clinic to a hospital, region or nation); this approach also has &QJ0;the potential to reduce duplication of effort and enhance acceptability. In certain areas of the world, local and regional adaptations of international evidence‐based practice guidelines have become mandatory in order for clinicians to care for the ill. When guidelines are adapted, local evidence is always considered, such as specific health questions relevant to local use; to specific needs, priorities, legislation, policies and resources; to scopes of practice within local health services.

A commonly used guideline tool is the Integrated Management of Childhood Illnesses (IMCI) guideline. This tool provides guidance for improving case management, assessment, provision of medication and counselling to reduce the under‐5 child mortality rate [9]. This guideline aims to equip professional nurses with the necessary skills to classify, manage, refer, and follow up with children, as well as counsel caregivers of under‐5 children suffering from one or more illnesses. Despite its proven effectiveness, the IMCI strategy has its challenges, such as access to diagnostics for assessment or medication for treatment, which can hinder full implementation. Professional nurses have also been reported to not optimally implement the strategy, through the omission of executing important steps of the guide [9].

This may stem not only from systemic constraints but also from a lack of contextual adaptation and limited insight into the guideline's application in real‐world scenarios. When guidelines are not adapted to local realities such as resource limitations, workflow constraints, or patient population characteristics, nurses may struggle to apply them fully. Given their role in rendering healthcare services, nurses possess valuable frontline perspectives on the feasibility, acceptability, and implementation of clinical recommendations. Their involvement in guideline adaptation is therefore essential to ensure that such tools are both usable and effective in practice.

### 1.2 Why Is It Important to Understand Nurses' Involvement?

Understanding the nurses' involvement in guideline development processes is important because nurses form the greater cohort of health professionals treating patients. Guidelines are beneficial in institutions that are nurse‐led to ensure safe, evidence‐based practice. Matlala et al. [10] indicate that most of the mistakes identified in guideline usage are due to poor knowledge and understanding of the guideline. Matlala et al. [10] have also advocated that people who are involved in the crafting of any policy or guideline usually have a better understanding when it comes to applying such a policy or using such a guideline. Active involvement of end‐users in the guideline development process has been shown to have important changes in practice [11]. For example, in France, it is mandatory for international guidelines to be adapted into the local and regional context [11]. The active involvement of end users in the adaptation process contributes to improved adherence to guidelines, thus improved treatment outcomes. Therefore, it is essential to explore and understand experiences that nurses have of participating in guideline development processes and any potential barriers to and facilitators of their involvement in guideline processes. While some literature has explored the involvement of healthcare professionals in guideline development, there is a notable gap in reviews that focus specifically on nurses. For example, a scoping review by De Leo et al. [7] explored the approaches to clinical guideline development, including the involvement of the multidisciplinary team. While this revealed the multi‐disciplinary team's comprehensive contribution, it did not focus on nurses specifically. This particular study is looking to explore the nurses' facilitators and barriers to the development and adaptation processes. Existing reviews tend to emphasize their role as end‐users of guidelines rather than active contributors to their development and contextual adaptation [12]. To date, no qualitative evidence synthesis (QES) has been identified that critically examines the experiences, contributions, and perspectives of nurses in the development and adaptation of clinical practice guidelines. This review seeks to fill that gap by synthesizing qualitative evidence on nurses' involvement in these processes. Understanding the nurses' involvement could contribute to enhanced insights into the challenges experienced when working with these guidelines. Nurses are known to be implementers of these guidelines; however, understanding the barriers and facilitators of their involvement could contribute towards developing ways that could be beneficial in the effective use of guidelines, thus ensuring improved treatment outcomes.

Qualitative evidence is important because it gives us an in‐depth understanding of a specific phenomenon. A QES offers a more powerful explanation of a phenomenon; it provides further enhanced insight that cannot be achieved by a single primary qualitative study [13]. To accurately address questions relating to guideline development processes, Flemming et al. [13] encourage the use of a QES due to its ability to provide understanding around complex, contextual factors (such as the involvement of multiple stakeholders). Additionally, the QES allows for integration of different qualitative studies, thus exploring diverse perspectives. A QES will be adopted as a method of enquiry, which could also uncover ways that are working in other countries that could be adopted and applied in other settings.

## 2 Review Question

The research question for this review is ‘What are the views, experiences, and perceived barriers and facilitators of nurses involved in the development or adaptation of clinical practice guidelines?’ In line with the SPIDER framework, this synthesis will focus on qualitative studies involving nurses (S), their participation in guideline development and adaptation (PI), using interviews or focus groups (D), to explore perceptions of engagement, challenges, and enabling factors (E), within a qualitative research paradigm (R).

## 3 Objectives

- To identify and synthesize studies exploring methods and processes for involving nurses in guideline development and adaptation.
- To explore the barriers to and facilitators (e.g., individual, interpersonal, organizational, systemic, etc.) of involving nurses in guideline development and adaptation.

## 4 Methods

### 4.1 Review Author Reflexivity

We will maintain a reflexive stance throughout the stages of the review process through regular team discussions. Our review team consists of authors with knowledge and experience in conducting systematic reviews and are from a variety of disciplinary backgrounds: child health nursing and primary care service delivery (L.V.), systematic reviews, including QES (S.C., B.S., T.K.), social sciences (B.S., S.C.), health systems and policy (B.S., T.K.) and clinical guideline methods (T.K.). To identify bias and challenge each member's interpretations, regular debriefing meetings will be held. Furthermore, we will keep a shared reflexive journal to record discussions, decisions, and reflections during key stages of the review (e.g., during data extraction, coding and theme development). Disagreements in interpretation will be resolved through team consensus, and all analytic decisions will be logged and audited. Based on our collective and individual experiences (as clinicians, academics and researchers), we anticipate the findings of our review to reveal a combination of organizational, professional and individual factors associated with nurse involvement in guideline processes. This structured reflexive approach aligns with the guidance of Downe et al. [14] and aims to promote transparency, trustworthiness, and rigour in the synthesis. We will, as a team, remain mindful of our presuppositions and support each other to minimize the risk of these skewing our analysis or the interpretation of our findings. The first author (L.V.) will keep a reflexive journal throughout the review process in which to document and reflect on progress and decisions made.

### 4.2 Criteria for Considering Studies for This Review

#### 4.2.1 Types of Studies

We will include studies that use both qualitative methods for data collection (e.g., focus group discussions, individual interviews, observation, diaries, document analysis, open‐ended survey questions) and qualitative methods for data analysis (e.g., thematic analysis, framework analysis, grounded theory). Mixed methods studies that use qualitative data collection and analysis methods will be included. However, we will exclude studies that collected data using qualitative methods but did not use qualitative methods for analysis (e.g., open‐ended survey questions where the response data are analyzed using descriptive statistics only). This decision is common practice for QES [15] and is based on the need for methodological coherence across included studies, as the review seeks to generate interpretive, in‐depth themes rather than descriptive or quantifiable outputs. We will, however, revisit their inclusion at the interpretive stage if critical gaps in the data emerge. Below are the types of studies that are eligible for inclusion in the review.

**Table:** 

Inclusion criteria.
Studies will be included if they:
(1) are published in any language, any geographic context, and published at anytime;
(2) are focusing on the involvement of nurses in the guideline development and adaptation;
(3) types of participants are nurses practicing in clinical settings;
(4) study design and methods should be clearly defined and include qualitative methods for data collection and analysis;
(5) report on factors (barriers and facilitators, context, views and experiences) affecting nurse involvement in guideline development and adaptation.

Studies that will be excluded are those of opinion pieces and commentary papers. Other studies that will be excluded are those that focus on the role of other health care practitioners (excluding nurses) in guideline development and adaptation, or if it is not possible to separate out the data of nurses from other health care practitioners.

#### 4.2.2 Topic of Interest

The topic of interest for this review is the identification and synthesis of studies exploring methods and processes for involving nurses in guideline development and adaptation. We are interested in studies exploring nurse involvement in the development and adaptation of guidelines, and the factors, processes and contexts that shape this involvement. In addition, we are also interested in understanding the existing barriers and facilitators that contribute towards their successful involvement in guideline development and adaptation. We will explore nurses' involvement in these guideline processes globally and possibly identify similarities and differences across global settings.

#### 4.2.3 Types of Participants

We will include studies that include the views and experiences of nurses. By nurse we mean a professional who is trained to care for the sick, who has successfully completed a programme of education approved by the nursing council of their country [16]. This includes nurse managers, registered nurses (sometimes referred to as a sister, professional nurse or head nurse) and nursing support staff (nursing assistants and enroled nurses).

### 4.3 Search Methods for Identification of Studies

#### 4.3.1 Electronic Searches

We will search for relevant literature in PubMed, CINAHL, Scopus, Medline, and PsycINFO. These databases are widely recognized for their broad indexing of nursing, medical, public health, and interdisciplinary literature, and are considered sufficient for capturing studies relevant to the scope of this review. An information specialist will assist with developing a search strategy in PubMed, which will be adapted to the other databases. The preliminary PubMed search strategy is presented in Appendix 1. We will not apply any limits on language, geographic location or publication date. We will apply a methodological filter for qualitative studies.

#### 4.3.2 Searching Other Resources

Grey literature such as academic dissertations (international and local) will be included provided those studies have obtained ethics approval. Experts in the field of interest will be contacted and consulted for additional studies they may be aware of and which meet inclusion criteria of the review. To further improve representativeness, we will screen selected grey literature sources, such as WHO IRIS, where applicable. Furthermore, we will do a backward reference search for all included studies.

### 4.4 Selection of Studies

We will import all search records into Covidence and remove duplicates. Two review authors (L.V., B.S., S.C. or T.K.) will independently screen the titles and abstracts against the eligibility criteria of the review. Covidence is a software package designed to prepare systematic reviews (www.covidence.org) for screening and study selection. If there are disagreements, the two authors will discuss, and if consensus is not reached, then a third reviewer will be involved if required. We will then retrieve the full‐text papers of the titles and abstracts that were deemed potentially eligible during the screening process. Two review authors (L.V., B.S., S.C. or T.K.) will independently assess the full text papers; disagreements will be resolved through discussion, and again, if necessary, with a third review author. A table with studies excluded during the full text stage will be included, with the reasons for exclusion specified. A PRISMA flowchart will be used to report on search results and the process of screening and selecting studies for inclusion.

### 4.5 Language Translation

Primary qualitative studies will be included irrespective of the language in which they were published. During the abstract screening stage, if the screened studies are published in other languages outside of English are identified as relevant, an open‐source software (e.g., Google Translate) will be used to carry out the initial translation. In cases where the screened abstract is not well‐understood after the software translation, the full text of the paper will be retrieved to enhance understanding of the study. Additionally, a translator will be sought should the open‐source software not translate accurately.

### 4.6 Sampling of Studies

Once the authors have identified all the eligible studies, they will then consider if the amount of data included is too large, as this could impact the quality of the QES. If this is found to be the case, the authors will sample from the identified studies [14, 17]. There is no formula for deciding how much data is ‘too much’ for inclusion in a QES, and therefore a judgement needs to be made that may vary across reviews and reviewers [18]. In line with current guidance, we will make this decision iteratively once we have identified and mapped key characteristics of all eligible studies [19]. That is, we will map the studies that meet the inclusion criteria by conducting data extraction of key study characteristics, including: date of publication, aim, setting, participants, and the amount and type of data (data richness and thickness) that is relevant for addressing the review objectives. For the latter, we will use a recently developed tool endorsed by Cochrane to assess the data richness and thickness of studies [20]. Based on this mapping, we will make a decision about whether to sample. This will depend on (1) how thick and rich the data relevant to answering the research question is; (2) the diversity of study participants and contexts and (3) whether the amount of data will hinder the quality of the analysis process [14, 19]. If we decide to sample, we will then employ maximum variation sampling, drawing on the approach developed by Ames et al. [17]. In line with this approach, we will apply a three‐step sampling framework which prioritizes studies representing a wide geographic spread (e.g., including high‐, middle‐ and low‐income countries), those comprising rich and thick data, and studies where the focus closely resembles the synthesis objectives.

### 4.7 Data Extraction

Data extraction will be based on the aim of the review, which is to identify and synthesize studies exploring methods and processes for involving nurses in guideline development and adaptation, and to explore the barriers and facilitators of involving nurses in guideline development and adaptation. A data extraction template will be used to extract the following contextual and methodological information from each study: Author surname, publication year, study design, source and type of publication (journal paper, abstract, conference proceeding, etc.), aim of the study, description of participants and the study setting (country, health facility, etc.), context (factors affecting nurses participation in guideline processes), method of data collection and analysis and related findings. The two authors will independently extract data from the included studies. If disagreement arises, a third reviewer's opinion will be asked to reach a consensus.

#### 4.7.1 Data Management, Analysis and Synthesis

The synthesis for this review will be guided by Thomas and Harden's thematic synthesis framework [21]. This framework combines and adapts approaches from both meta‐ethnography and grounded theory [22]. It involves line‐by‐line coding of study findings, developing descriptive themes and generating analytical themes; this is the three‐step process to be followed by the review authors. This approach shares characteristics with adaptations of meta‐ethnography, in that the analytical themes are comparable to ‘third order interpretations’ and that the development of descriptive and analytical themes using coding invokes reciprocal ‘translation’. It also shares much with grounded theory, in that the approach is inductive and themes are developed using a ‘constant comparison’ method [22]. This synthesis method has been chosen as it allows for the inductive identification and development of themes that reflect the included studies' data. Two review authors (L.V. and B.S.) will independently code the data from the included studies. We will ensure internal validity is maintained through blinded double coding. We will compare and discuss coding interpretations, resolve discrepancies through dialogue, and refine the coding framework before applying it to the full data set. The other authors will check the codes and compare the codes for consistency.

ATLAS. Ti will be used as the data analysis software tool for organizing the extracted data, assisting with the coding, and linking themes back to their original sources. Additionally, this is to ensure that the recommended methodological transparency of QES is supported.

### 4.8 Assessing the Methodological Limitations of Included Studies

Munthe‐Kaas et al. [18] define methodological limitations as the extent to which there are concerns about the design or how the included primary study was conducted. When the primary studies that have impacted the review finding are assessed for methodological limitations, there could be less confidence that the review finding actually reflects the phenomenon of interest. The purpose is not to judge the standard of methodological quality, but rather to indicate concerns where methodological limitations are identified as being serious enough to compromise the confidence of the review finding.

The Critical Appraisal Skills Programme (CASP) tool will be the tool used in this review to assess for methodological limitations. Each included study will be assessed independently by the two review authors on the extent to which each quality criterion is met. Should there be a disagreement between the two review authors, a discussion will be had, and a third reviewer will be involved if the disagreement is unable to be resolved. The CASP tool includes the following 10 questions, which will be used to assess the methodological limitations.

- Was there a clear statement of the aims of the research?
- Is a qualitative methodology appropriate?
- Was the research design appropriate to address the aims of the research?
- Was the recruitment strategy appropriate to the aims of the research?
- Was the data collected in a way that addressed the research issue?
- Has the relationship between researcher and participants been adequately considered?
- Have ethical issues been taken into consideration?
- Was the data analysis sufficiently rigorous?
- Is there a clear statement of findings?
- Is the research valuable?

Given the nature of the study, the Critical Interpretive Synthesis (CIS) checklist will be used as a complementary tool that supports the fuller assessment of conceptual rigour [23]. Using the criteria of the CIS checklist will allow us to assess the theoretical contribution, depth of interpretation and conceptual clarity. The CIS‐informed criteria will include:

- Conceptual clarity: Does the study present clearly defined concepts or theoretical constructs?
- Interpretive depth: Does the analysis move beyond &QJ0;simple description to offer new insights or theoretical explanations?
- Theoretical contribution: Does the study challenge, extend, or refine existing theory or practice?
- Reflexivity: Does the study demonstrate critical reflection on the researcher's influence in data collection and analysis?
- Relevance to synthesis: Does the study offer transferable insights that can contribute meaningfully to the development of new themes or theories?

A pilot will be conducted on three included studies to assess the feasibility of using this tool and to assess if it is workable for this review. Assessments of methodological limitations will be presented in a table.

### 4.9 Assessing Our Confidence in the Review Findings

#### 4.9.1 GRADE‑CERQual

Three review authors (L.V., B.S., S.C.) will use the GRADE‐CERQual (Confidence in the Evidence from Reviews of Qualitative research) approach to assess our confidence in each review finding [19]. CERQual assesses confidence in the evidence, based on the following four key components.

1. Methodological limitations of included studies: the extent to which there are concerns about the design or conduct of the primary studies that contributed evidence to an individual review finding.
2. Coherence of the review finding: an assessment of how clear and cogent the fit is between the data from the primary studies and a review finding that synthesizes those data. By cogent, we mean well‐supported or compelling.
3. Adequacy of the data contributing to a review finding: an overall determination of the degree of richness and quantity of data supporting a review finding.
4. Relevance of the included studies to the review question: the extent to which the body of evidence from the primary studies supporting a review finding is applicable to the context (perspective or population, phenomenon of interest, setting) specified in the review question.

The assessment of each finding will be carried out independently by the two reviewers, with final judgements based on discussion and consensus. We will resolve any disagreements through dialogue, and if necessary, consultation with additional review authors. After assessing each of the four components, we will make a judgement about the overall confidence in the evidence supporting the review finding. We will judge confidence as high, moderate, low, or very low. All findings start as high confidence and will then be graded down if there are important concerns regarding any of the CERQual components.

#### 4.9.2 Summary of Qualitative Findings Table and Evidence Profile

The findings of the review and the assessment of confidence in the findings will be presented in the Summary of Qualitative Findings table to ensure understanding and easy interpretation. The table will be displayed in a structured manner. It will display each review finding, the reference, and the explanation of the assessment of confidence as guided by the GRADE‐CERQual approach [24]. To ensure transparency and completeness in the reporting of QES methods, this review will adhere to the ENTREQ (Enhancing Transparency in Reporting the Synthesis of Qualitative Research) checklist. The ENTREQ framework will guide the documentation and reporting of key synthesis stages, including search strategy, study selection, data extraction, and analytical processes. A completed ENTREQ checklist will be submitted as Supporting Information S1.

### 4.10 Proposed Timeline and Review Updates

This QES will follow a structured, staged process from protocol finalization to reporting. To maintain the relevance and currency of the findings, the search strategy will be designed for repeatability and may be updated annually, particularly if the review extends over an 18‐month period or more. This aligns with good practice recommendations for living or semi‐updated qualitative syntheses. A final search update will be considered before data synthesis if a significant delay occurs between the initial search and the analysis phase. The following tentative timeline outlines estimated durations for each review phase, assuming an 18‐month total review period. Specific dates are not provided to retain flexibility.

**Table:** 

Review phase	Estimated duration
Protocol finalization and team calibration	1 month
Literature search and study selection	2–3 months
Data extraction and methodological appraisal (CASP + CIS)	2–3 months
Thematic synthesis and development of analytical themes	3–4 months
CERQual assessment and synthesis of findings	3 months
Drafting and revising the manuscript for submission	2–3 months
Optional search update before synthesis (if applicable)	Ongoing/as necessary

## Author Contributions

**Linda Velapi:** conceptualization, methodology, writing – original draft, writing – review and editing. **Sara Cooper:** supervision, methodology, conceptualization. **Tamara Kredo:** conceptualization, supervision, funding acquisition. **Bey Schmidt:** methodology, supervision, conceptualization.

## Conflicts of Interest

The authors declare no conflicts of interest.

## Supporting information

ENTREQ Checklist for QES Protocol.

## Data Availability

The data that supports the findings of this study will be available within the article and its supporting material.

## Appendix A

### A.1

See Table A1.

**Table A1:** Example of search strategy (PubMed).

Search number	Query	Sort by	Filters	Search details	Results	Time
4	((‘Guideline’ [Publication Type] OR ‘Practice Guideline’ [Publication Type]) OR (‘Clinical Protocols’[Mesh] OR ‘Practice Guideline’ [Publication Type])) AND (‘Nurses’[Mesh] OR ‘Nurses, Community Health’[Mesh] OR ‘Nurses, Public Health’[Mesh] OR ‘Nurses, Pediatric’[Mesh] OR ‘Nurse Practitioners’[Mesh] OR ‘Nurse Clinicians’[Mesh])			(‘Guideline’[Publication Type] OR ‘Practice Guideline’[Publication Type] OR (‘Clinical Protocols’[MeSH Terms] OR ‘Practice Guideline’[Publication Type])) AND (‘Nurses’[MeSH Terms] OR ‘nurses, community health’[MeSH Terms] OR ‘nurses, public health’[MeSH Terms] OR ‘nurses, pediatric’[MeSH Terms] OR ‘Nurse Practitioners’[MeSH Terms] OR ‘Nurse Clinicians’[MeSH Terms])	515	11:00:35
3	‘Clinical Protocols’[Mesh] OR ‘Practice Guideline’ [Publication Type]	Most recent		‘Clinical Protocols’[MeSH Terms] OR ‘Practice Guideline’[Publication Type]	231,815	10:34:38
2	‘Guideline’ [Publication Type] OR ‘Practice Guideline’ [Publication Type]	Most recent		‘Guideline’[Publication Type] OR ‘Practice Guideline’[Publication Type]	40,669	10:33:34
1	‘Nurses’[Mesh] OR ‘Nurses, Community Health’[Mesh] OR ‘Nurses, Public Health’[Mesh] OR ‘Nurses, Pediatric’[Mesh] OR ‘Nurse Practitioners’[Mesh] OR ‘Nurse Clinicians’[Mesh]	Most recent		‘Nurses’[MeSH Terms] OR ‘nurses, community health’[MeSH Terms] OR ‘nurses, public health’[MeSH Terms] OR ‘nurses, pediatric’[MeSH Terms] OR ‘Nurse Practitioners’[MeSH Terms] OR ‘Nurse Clinicians’[MeSH Terms]	102,315	10:28:39
