# Supplementary material for: Barriers and Facilitators of Nurses' Involvement in Guideline Development and Adaptation: A Qualitative Evidence Synthesis Protocol
Source: Clin Public Health Guidel. 2025 Sep 20;2(4):e70044. doi: 10.1002/gin2.70044 (PMC13563405; doi:10.1002/gin2.70044)
Supplement: Supplementary file 1 [file GIN2-2-e70044-s001.pdf]

***Title**: Barriers and facilitators of nurses' involvement in guideline development and adaptation: a qualitative evidence synthesis protocol.*

**The ENTREQ Checklist (QES Protocol)**

| Item | Guide / Description | Reported on (page/section) | Notes / Status |
|---|---|---|---|
| **Aim** | State the research question the synthesis addresses | Abstract p.1; Review Question section p.6 | Stated clearly |
| **Synthesis methodology** | Identify the synthesis methodology or theoretical framework; describe rationale | Methods p.11 | Thematic synthesis (Thomas & Harden) |
| **Approach to searching** | Indicate whether pre-planned or iterative | Methods p.8-9 | Pre-determined strategy; annual updates possible |
| **Inclusion criteria** | Specify inclusion/exclusion criteria | Methods p.7-8 (Criteria for considering studies); Table 1 | Clearly defined |
| **Data sources** | Describe information sources used | Methods p.8-9 | PubMed, CINAHL, Scopus, Medline, PsycINFO, grey lit |
| **Electronic search strategy** | Describe electronic search terms/filters | Appendix 1 p.18 | PubMed example included |
| **Study screening methods** | Describe screening and sifting process | Methods p.10 | Covidence, dual independent reviewers |
| **Study characteristics** | Present included study characteristics | Not applicable (protocol) | Future stage |
| **Study selection results** | Identify numbers screened/excluded, reasons | Not applicable (protocol) | Future stage |
| **Rationale for appraisal** | Describe rationale and approach for appraising included studies | Methods p.11-12 | CASP + CIS planned |
| **Appraisal items** | State tools/frameworks used to appraise | Methods p.11-12 | CASP + CIS checklist |

| | | | |
|---|---|---|---|
| **Appraisal process** | Indicate whether the appraisal is conducted independently by more than one reviewer and if consensus was required. | Methods p.11-12 | Independent assessment by 2 reviewers, consensus with third |
| **Appraisal results** | Present results of the quality assessment and indicate which articles, if any, were weighted/excluded based on the assessment and give the rationale. | Not applicable (protocol) | Future stage |
| **Data extraction** | Indicate which sections of the primary studies were analysed and how were the data extracted from the primary studies? | Methods p.10-11 | Data extraction template, dual independent |
| **Software** | State computer software to be used | Methods p.11 | ATLAS.ti |
| **Number of reviewers** | Identify who will be involved in coding/analysis | Methods p.11 | LV & BS, with others checking |
| **Coding** | Describe coding process | Methods p.11 | Line by line coding, thematic synthesis |
| **Study comparison** | Describe comparisons within/across studies | Methods p.11 | Constant comparison method |
| **Derivation of themes** | Explain inductive/deductive derivation | Methods p.11 | Inductive, constant comparison |
| **Quotations** | Provide quotations to illustrate themes | Not applicable (protocol) | Future stage |
| **Synthesis output** | Present rich, compelling results beyond summary | Not applicable (protocol) | Future stage |
